# Implementing the transvaginal ultrasound simulation training (TRUSST) programme for obstetric registrars

**DOI:** 10.1186/s41077-020-00152-y

**Published:** 2021-01-12

**Authors:** Sally Byford, Sarah Janssens, Rachel Cook

**Affiliations:** 1 Mater Health, Mater Hospital, Brisbane, Australia; 2 Mater Education, Mater Hospital, Brisbane, Australia

**Keywords:** Transvaginal ultrasound, Simulation, ScanTrainer, Education, Early pregnancy

## Abstract

**Background:**

Transvaginal ultrasound (TVUS) training opportunities are limited due to its intimate nature; however, TVUS is an important component of early pregnancy assessment. Simulation can bridge this learning gap.

**Aim:**

To describe and measure the effect of a transvaginal ultrasound simulation programme for obstetric registrars.

**Materials and methods:**

The transvaginal ultrasound simulation training (TRUSST) curriculum consisted of supported practice using virtual reality transvaginal simulators (ScanTrainer, Medaphor) and communication skills training to assist obstetric registrars in obtaining required competencies to accurately and holistically care for women with early pregnancy complications.

Trainee experience of live transvaginal scanning was evaluated with a questionnaire. Programme evaluation was by pre-post self-reported confidence level and objective pre-post training assessment using Objective Structured Assessment of Ultrasound Skills (OSAUS) and modified Royal Australian and New Zealand College of Obstetrics and Gynaecology assessment scores. Quantitative data was compared using paired *t* tests.

**Results:**

Fifteen obstetric registrars completed the programme. Numbers of performed live transvaginal ultrasound by trainees were low. Participants reported an increase in confidence level in performing a TVUS following training: mean pre score 1.6/5, mean post score 3/5. Objective assessments improved significantly across both OSAUS and RANZCOG scores following training; mean improvement scores 7.6 points (95% CI 6.2–8.9, *p* < 0.05) and 32.5 (95% CI 26.4–38.6, *p* < 0.05) respectively. It was noted that scores for a systematic approach and documentation were most improved: 1.9 (95% CI 1.4–2.5, *p* < 0.05) and 2.1 (95% CI 1.5–2.7, *p* < 0.05) respectively.

**Conclusion:**

The implementation of a simulation-based training curriculum resulted in improved confidence and ability in TVUS scanning, especially with regard to a systematic approach and documentation.

## Introduction

Diagnostic delay regarding pregnancy viability is challenging for women and can adversely affect their perception of hospital care. The introduction of early pregnancy assessment units provides continuity of care and patient satisfaction [1]. When skilled clinicians are available, point of care TVUS results in a lower rate of admissions from 40.3 to 17.1% and can alter clinical management in 54.1% of patients [2]. Despite the Royal Australian and New Zealand College of Obstetrics and Gynaecology (RANZCOG) introducing ultrasound competencies for new trainees since 2016, many existing trainees have had minimal training in transvaginal ultrasound (TVUS).

Opportunities to learn transvaginal scanning are fewer than transabdominal scanning due to its intimate nature. Supplementing training with simulation may result in faster upskilling and provide greater standardisation of the learning experience. The demonstrated benefits of simulation training include improved confidence [3, 4], a better learning experience [5] and improved performance—both self-assessed [3] as well as objectively assessed [6]. From a service-based point of view, TVUS simulation training reduces the supervision and repetition of scans required [3, 6] therefore presumably makes a more efficient and cost-effective service. Additionally, objective, automated measures of performance available on virtual reality (VR) simulators may provide a reproducible assessment of base-level psychomotor competency required prior to live scanning, similar to the role of VR laparoscopic trainers [7]. Grantcharov and Reznick [8] demonstrated the importance of pre-patient training following acquisition of cognitive knowledge when teaching procedural skills.

The objective of this project was to assess trainee live TVUS experience and evaluate the introduction of the transvaginal ultrasound simulation training (TRUSST) programme, a simulation-based programme for TVUS scanning by obstetric registrars. This programme was developed to address limited opportunities, trainee’s lack of confidence and a lack of standardised skill development in TVUS.

## Materials and methods

### Participants/setting

Obstetric registrars training at a tertiary level hospital in 2018 were invited to participate in the programme during rostered education time. The hospital provides a 24/7 Early Pregnancy Assessment service and assesses on average 17 patients with early pregnancy complications daily. Consent was obtained from all participants and approval for the programme was granted by the Mater Health Service Human Research Ethics committee (Reference HREC/18/MHS/4).

### Curriculum development

A modular curriculum was developed with input from our Sonography Educator, Clinical Simulation Director, Maternal Fetal Medicine consultants and the Bereavement Service. A virtual reality (VR) transvaginal simulator (ScanTrainer, Medaphor, Cardiff, UK) was used to teach the skills required to diagnose early pregnancy complications using TVUS. The programme consisted of an online video tutorial and face-to-face sessions as well as supervised practice and unlimited independent practice using ScanTrainers. Key learning objectives included demonstration of appropriate communication skills for counselling and consenting patients, using a standard framework for scanning and obtaining required images in early pregnancy assessment. Registrars were required to reach a pre-set level of proficiency prior to gaining further experience through supervised scanning on patients—this was determined by the supervisors subjectively ensuring that participants had reached a level to minimise discomfort and inefficiency with patients.

A short online learning video ensured baseline knowledge of scanning technique covering ultrasound physics, views and probe care. The initial face-to-face 3-h session discussed possible presentations of early pregnancy complications, introducing the content and relevance of TVUS skills. This promoted a scaffolding approach and addressed various levels of Miller’s Pyramid [9] whilst encouraging participant interaction and active learning. Next, Malouf’s [10] technique of “tell-show-do-review” was used, with learners first observing a TVUS assessment, it was then deconstructed and explained in separate steps before practising those steps. A visual aid was provided as a framework to prompt each step of the scan, aiming to assist with differing learning styles [11]. The second session utilised role play to practice and refine techniques for consenting patients for intimate procedures and counselling them for inconclusive and bad news scenarios with input and feedback from a bereavement counsellor. The largest component was hands-on supervised practice on a ScanTrainer with a supervisor, for learners to gain the procedural skills required and promote practice fixation. Time and practice are required to develop competence and autonomy [12] and simulation provided a risk and emotion-free environment to achieve conditioning [13]. The trainees had exposure to the ScanTrainer modules as well as additional uploaded de-identified real patient sweeps that the sonography educator created.

### Measures

Participants completed a pre-post survey which collected demographic data and the number of TVUS performed under supervision and independently. Pre-post Likert scales evaluated trainee confidence with assessing the need for a TVUS, performing a TVUS, making a management plan based on a TVUS report, as well as their comfort level counselling patients with early pregnancy loss and inconclusive results. Free text comments regarding the programme were collected.

Participants’ scanning skills were assessed on the simulator by trained supervisors using an uploaded standard early pregnancy scan. Comparable new scans were used in the post-training assessment to ensure participants had not had prior exposure to, or familiarity with, the test cases. Raters used the validated Objective Structured Assessment of Ultrasound Skills (OSAUS) score [14] but with three components removed as they were deemed not relevant by the sonography educator to the assessed scan. OSAUS components include image optimisation, systematic examination, interpretation of images and documentation of examination, with a total score out of 20. The locally relevant RANZCOG first trimester transvaginal ultrasound assessment of procedural and surgical skills (APSS) sheet was out of a total of 72 as gestational sac measurement was not applicable to the assessed scan and fetal heart motion was unable to be measured on uploaded images. Two tools are used to ensure that results are both relevant to a validated score as well as local training requirements.

### Statistics

Data was analysed using IBM SPSS Statistics Version 24. Data was tested for normal distribution using Shapiro-Wilk method, and differences between the pre-training and post-training groups were assessed using a paired samples *t* test.

## Results

Fifteen obstetric registrars completed the programme with demographic characteristics shown in Table 1. Trainees spent between 2 and 21 h on the ScanTrainer. The number of transvaginal scans, both supervised and unsupervised, performed by the registrars pre- and post-training is shown in Table 2. Scan numbers highlight a deficiency in current trainee experience with transvaginal ultrasound scanning, with only one trainee having performed over 10 TVUS. RANZCOG recommend a minimum of 80 h of scanning within the first 2 years of training.

**Table 1:** Participant characteristics

Participant characteristic (***n*** = 15)		
**Age (mean (range) years)**		32.9 (26–41)
**Gender**	Male	2 (13%)
	Female	13 (87%)
**Dominant hand**	Right	13 (87%)
	Left	2 (13%)
**Training year**	Non-training registrar	3 (20%)
	1	2 (13%)
	2	2 (13%)
	3	1 (7%)
	4	3 (20%)
	5	1 (7%)
	6	3 (20%)

**Table 2:** Real patient scanning experience pre- and post-training

		Number of participants (*n* = 15)	
		Pre-training	Post-training
Number of supervised TV scans	0	4	2
	1–5	5	4
	6–10	5	1
	11–25	1	3
	26–50	0	4
	> 50	0	1
Number of unsupervised TV scans	0	12	10
	1–5	2	2
	6–10	0	0
	11–25	1	2
	26–50	0	2
	> 50	0	0

**Fig. 1:**
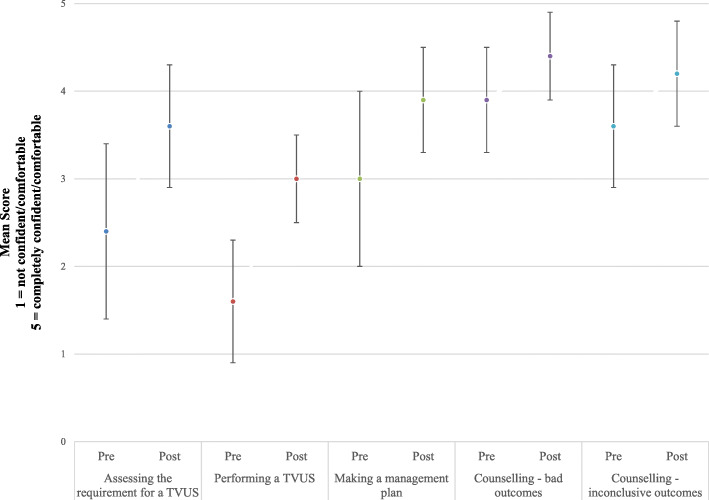
Self-evaluation of confidence (*n* = 15): mean score with 1.0 SD

Participants reported an increase in confidence or comfort across all five evaluation questions (Fig. 1), with an average increase of 1.4 point (95% CI 0.9–1.9, *p* < 0.05) on the 5-point Likert scale in confidence performing a TVUS. The participant comments suggest that the provision of a “structure to perform a scan” and a “standardisation of the expected skill level”, with “personal supervised support and guidance”, contributed to this improvement. Comments for suggested improvements recurrently consisted of “more practice scanning cases”, “more protected time required” and “more access to then progress skills to live patient scanning”.

**Fig. 2:**
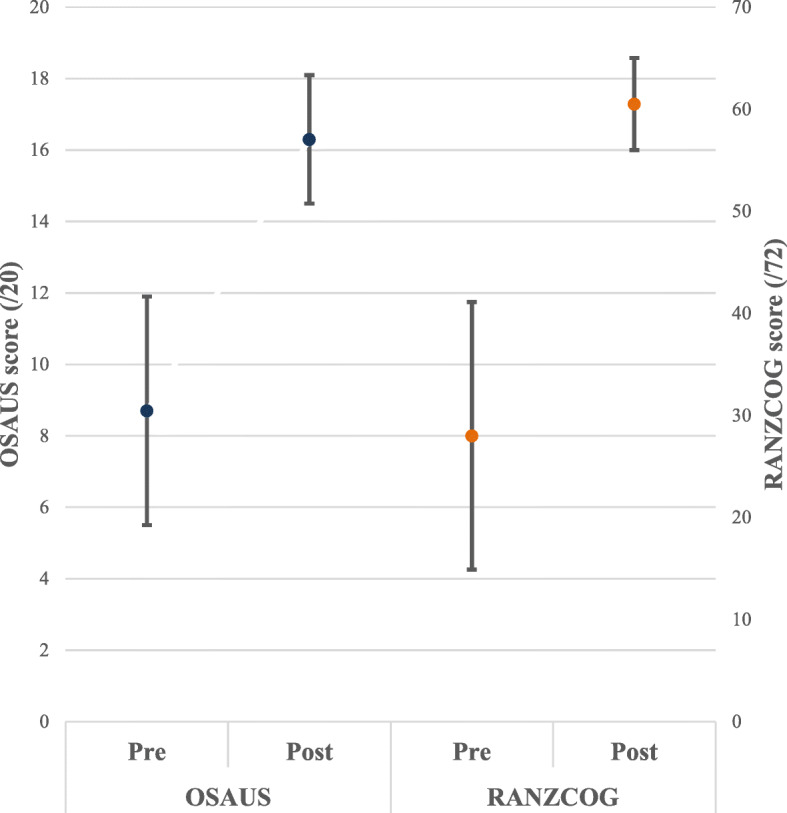
Mean test scores pre- and post-training using the OSAUS and RANZCOG scores with 1.0 SD

Participants demonstrated a significant improvement in the pre- and post-training assessments in both assessment tools. On the OSAUS score (/20), the participants’ mean score was 8.7/20 (SD 3.2) pre-training and 16.3 (SD 1.8) post-training—mean improvement score 7.6 points (95% CI 6.2–8.9, *p* < 0.05), whilst mean RANZCOG scores (/72) increased from 28 (SD 13.1) to 60.5 (SD 4.5) pre- to post-training, mean improvement score 32.5 points (95% CI 26.4–38.6, *p* < 0.05). Figure 2 demonstrates that not only did the mean score increase but the variability in score also reduced when using either score. When the OSAUS score was broken down into the individual criteria—each being scored between 1 and 5, with 5 being consistently performing the component accurately—systematic approach and documentation were most improved with mean score improvements of 1.9 (95% CI 1.4–2.5, *p* < 0.05) and 2.1 (95% CI 1.5–2.7, *p* < 0.05) respectively.

## Discussion

The TRUSST programme provided trainees with an opportunity to improve TVUS confidence and skills with a structured learning approach.

Reported live scan numbers were low, highlighting a deficiency in current trainee experience with TVUS. Six registrars had rostered time in Maternity Fetal Medicine accounting for the higher number of scans for some registrars. However, two thirds of participants had still only performed fewer than 25 supervised TVUS despite a busy Early Pregnancy Assessment Unit. The International Society of Ultrasound in Obstetrics and Gynaecology [15] recommend that a minimum of 100 transvaginal scans are required prior to proficiency, which may not be achievable during RANZCOG training. Potential lack of TVUS opportunities is not an issue isolated to this specific hospital with participants likely to be representative of RANZCOG trainees in general as they included a range of levels and had been to a variety of training hospitals throughout Queensland prior to rotation to our unit. While simulation programmes such as TRUSST have the potential to assist in meeting the gap in skill development, low number of scans performed post-training would suggest further investigation into barriers to accessing live training is required.

One such barrier may be trainee confidence, and participants reported poor confidence levels prior to training. The TRUSST programme resulted in improved confidence levels in participants performing a TVUS, and while confidence does not equate to competence, it can be a barrier to performing procedures, especially intimate ones [16].

Although our study demonstrated improvement in scanning skill on the simulator, Tolsgaard et al. [17] demonstrated a sustained improvement in scanning abilities on live patients 2 months following simulation training with clinical experience compared to clinical experience alone.

Consistent with demonstrated improvements in systematic approach scores, the scanning framework visual aid was frequently commented on as highly beneficial component of the programme in addition to the opportunity to use the ScanTrainer. It was also noted subjectively that this improved systematic approach reduced the time taken to scan and conclude the findings. Time taken to scan was not formally recorded but could be included in future studies, not to encourage fast scans, but to emphasise the importance of a systematic approach for efficiency, accuracy and patient comfort. Similar improvements have been seen with laparoscopic VR simulators which have been shown to reduce operating time, increase accuracy and decrease errors [18].

Tolsgaard et al. [17] suggest that TVUS simulation is applicable to not only psychomotor skill development but may also be used for competency assessment. Simulator assessment of competency prior to live performance is common across many procedural skills and has led to improvements in patient care [19]. Competency assessment may be an important area of focus for future research and curriculum development in TVUS skills. To obtain mastery of point of care ultrasound Lewiss, Hoffmann, Beaulieu, and Phelan [20] suggest that competence is required in three components: image acquisition, interpretation and integration into the clinical picture. Dynamic simulators such as ScanTrainer allow for both normal and abnormal findings, and the use of M-Mode, and may be maximally beneficial in developing skills in image acquisition [20]. While potentially superior to static simulators in this regard, there still remains a lack of variety in its standard cases, limiting potential learning in image interpretation and implications for clinical management. Access to a Cloud-based image library can expand case variety; however, many cases are not necessarily specific to the needs of obstetric trainees. In ongoing curriculum development, the addition of further virtual patients by uploaded de-identified image sweeps from real patients may provide added case exposure, improving opportunities for trainee to engage in clinical interpretation, critical thinking and self-analysis [21].

Limitations in this study include the small number of participants, and that it was a single-centre study in a tertiary hospital with access to a ScanTrainer so may not be applicable to other centres. These are common limitations in studies assessing the development of technical skills [22]. It also does not take into consideration the cost effectiveness of the training with regard to department efficiency and patient care; this could be examined in a further study. We acknowledge that by reducing the components on both the OSAUS and RANZCOG scores, the validity of these scores may be compromised, but in terms of assessing scanning skills on a simulator, these were felt necessary. Perhaps a full scenario could be used in future training or studies using a hybrid model with a standardised patient and the simulator combined. Real patients would be preferable; however, especially for pre-training, this is unethical and unfair on these patients with such an emotional and intimate situation. Lastly, real patient scanning skills have not been assessed within this study, although have been demonstrated with VR TVS previously [6, 23].

## Conclusions

In conclusion, the TRUSST programme resulted in significant improvements in simulated scanning using quantitative measures as well as improved participant confidence in performing a TVUS, and counselling patients with early pregnancy complication. This simulation programme can provide a standardised training experience in an area where live patient exposure is difficult to access. Future improvements to the curriculum should aim to increase the variety of full pelvic scan cases to increase content validity supported by protected training time for simulated training. Further studies are required to fully assess the impact of the TRUSST programme on patient care and department efficiency.

## Data Availability

The datasets during and/or analysed during the current study available from the corresponding author on reasonable request.

## Abbreviations

TVUS: Transvaginal ultrasound
TRUSST: Transvaginal ultrasound simulation training
OSAUS: Objective Structured Assessment of Ultrasound Skills
RANZCOG: Royal Australian and New Zealand College of Obstetrics and Gynaecology
